# A Technology for Anti-Thrombogenic Drug Coating of Small-Diameter Biodegradable Vascular Prostheses

**DOI:** 10.17691/stm2020.12.6.01

**Published:** 2020-12-28

**Authors:** L.V. Antonova, E.O. Krivkina, M.A. Rezvova, V.V. Sevostyanova, V.O. Tkachenko, T.V. Glushkova, T.N. Akentyeva, Yu.A. Kudryavtseva, L.S. Barbarash

**Affiliations:** Head of the Laboratory of Cell Technologies, Department of Experimental Medicine; Research Institute for Complex Issues of Cardiovascular Diseases, 6 Sosnovy Blvd, Kemerovo, 650002, Russia;; Junior Researcher, Laboratory of Cell Technologies, Department of Experimental Medicine; Research Institute for Complex Issues of Cardiovascular Diseases, 6 Sosnovy Blvd, Kemerovo, 650002, Russia;; Junior Researcher, Laboratory of New Biomaterials, Department of Experimental Medicine; Research Institute for Complex Issues of Cardiovascular Diseases, 6 Sosnovy Blvd, Kemerovo, 650002, Russia;; Researcher, Laboratory of Cell Technologies, Department of Experimental Medicine; Research Institute for Complex Issues of Cardiovascular Diseases, 6 Sosnovy Blvd, Kemerovo, 650002, Russia;; Senior Researcher; Budker Institute of Nuclear Physics, Siberian Branch of the Russian Academy of Sciences, 11 Acad. Lavrentieva Avenue, Novosibirsk, 630090, Russia; Researcher, Laboratory of New Biomaterials, Department of Experimental Medicine; Research Institute for Complex Issues of Cardiovascular Diseases, 6 Sosnovy Blvd, Kemerovo, 650002, Russia;; Junior Researcher, Laboratory of New Biomaterials, Department of Experimental Medicine; Research Institute for Complex Issues of Cardiovascular Diseases, 6 Sosnovy Blvd, Kemerovo, 650002, Russia;; Head of the Department of Experimental Medicine; Research Institute for Complex Issues of Cardiovascular Diseases, 6 Sosnovy Blvd, Kemerovo, 650002, Russia;; Professor, Academician of the Russian Academy of Sciences, Chief Researcher Research Institute for Complex Issues of Cardiovascular Diseases, 6 Sosnovy Blvd, Kemerovo, 650002, Russia;

**Keywords:** biodegradable vascular prostheses, small-diameter grafts, anti-thrombogenic drug coating, hemocompatibility

## Abstract

**The aim of the study** was to develop a technology for anti-thrombogenic drug coating of biodegradable porous scaffolds and to evaluate the physicomechanical and hemocompatible properties of functionally active vascular prostheses with and without a drug coating.

**Materials and Methods.:**

Vascular prostheses from polyhydroxybutyrate/valerate and polycaprolactone with the incorporated vascular endothelial growth factor, the main fibroblast growth factor, and the chemoattractant SDF-1α were made by emulsion electrospinning. Additional surface modification of the prostheses was carried out by forming a hydrogel coating of polyvinylpyrrolidone capable of binding drugs as a result of complexation. Unfractionated heparin and iloprost were used as anti-thrombogenic drugs.

**Results.:**

We show that after the modification of vascular prostheses with heparin and iloprost, a 5.8-fold increase in the Young’s modulus value was noted, which indicated a greater stiffness of these grafts compared to the unmodified controls. Platelet aggregation on the surface of heparin + iloprost coated vascular prostheses was 3.3 times less than that with the unmodified controls, and 1.8 times less compared to intact platelet-rich plasma. The surface of vascular prostheses with heparin and iloprost was resistant to adhesion of platelets and blood proteins.

**Conclusion.:**

Drug (unfractionated heparin and iloprost) coating of the surface of biodegradable prostheses significantly improved the anti-thrombogenic properties of these grafts but contributed to the increased stiffness of the prostheses.

## Introduction

Stenosis and occlusion of blood vessels are among the major causes and risk factors of cardiovascular diseases [1]. Surgical treatment of these conditions is based on replacing the damaged blood vessel with a section of an autologous vessel or with a vascular prosthesis [1]. The role of autologous veins and arteries as potential shunts may be limited due to previous surgeries on these vessels, progressive atherosclerosis, and other vascular diseases. Synthetic prostheses with a diameter of less than 6 mm, which are currently used in clinical practice, are subject to thrombosis and a risk of neointimal hyperplasia in the long-term postoperative period; therefore, such prostheses are rarely used to replace small-caliber arteries and veins [2]. To solve these problems, manufacturers offer synthetic prostheses coated with an anti-thrombogenic drug, which in some cases improves the patency of prostheses with a diameter of 6 mm [3]. However, an effective vascular implant with a diameter of less than 4 mm is yet to be developed.

Vascular tissue engineering is one of the promising ways of developing blood vessel prostheses [4]. There are various approaches to tissue engineering of blood vessels, but all of them are aimed at creating a vascular implant with a structure similar to that of a natural artery that would stay functional for a long time after surgery. The basis of such a vascular prosthesis is an artificial tubular matrix, most often made of biodegradable natural and/or synthetic polymers with high biocompatibility [5]. The matrix consists of a scaffold populated by autologous cells *in vitro* or *in situ*. Populating the matrix with the patient’s cells *in vitro* is a very laborious, time-consuming, and expensive process that is difficult to implement in a clinic, especially during emergency operations. In contrast, the *in situ* matrix colonization by cells at the site of implantation occurs as a natural process of implant bio-remodeling. The addition of biologically active substances, such as growth factors, chemokines, interleukins, amino acids, and others, into the structure of the implant, allows for their subsequent prolonged-release thus mimicking natural biochemical signals and stimulating regeneration of all structural layers of the vascular tissue, including the endothelium [6, 7].

Despite the promising perspective and seeming simplicity of this approach, the existing prostheses have a number of disadvantages, in particular, eventual thrombosis. To tackle this obstacle, one needs to enhance the anti-thrombogenic properties of tissue-engineered small-diameter vascular prostheses.

Among the suggested approaches is the formation of a thromboresistant inner surface to prevent implant thrombosis in the early postoperative period [7–10].

Earlier, at the Research Institute for Complex Issues of Cardiovascular Diseases (Kemerovo, Russia), a biodegradable small-diameter vascular prosthesis based on polyhydroxybutyrate/valerate (PHBV) and polycaprolactone (PCL) was developed and tested *in vitro* and *in vivo*; notably, a mixture of pro-angiogenesis factors (GFmix) was incorporated layer-by-layer into this polymer tubular scaffold by emulsion electrospinning [11–13]. In the final product, vascular endothelial growth factor (VEGF) is incorporated into the inner third of the prosthesis wall, and a combination of the basic fibroblast growth factor (bFGF) and the chemoattractant SDF-1α is incorporated into the outer 2/3 of the prosthesis wall. After the implantation into the rat abdominal aorta, PHBV/PCL grafts (with a diameter of 1.5 mm) containing the GFmix complex (VEGF + bFGF + SDF-1α) were shown to actively attract mature and progenitor cells from the bloodstream and surrounding tissues. From these cells, a layer of mature endothelial cells, a full-fledged neointima, and a layer of smooth muscle cells were generated after 3 months; collagens of types I and IV were detected as well [12]. The final (12 months after surgery) patency of these PHBV/PCL/GFmix prostheses with a diameter of 1.5 mm was 93.3% [12].

However, in a large animal model mimicking the human physiology, the highly porous surface of these grafts tended to provoke thrombosis. Therefore, additional anti-thrombogenic coating was needed to cover the surface of functionally active small-diameter vascular prostheses.

**The aim of the study** is to develop a technology for anti-thrombogenic drug coating of biodegradable porous scaffolds and to evaluate the physicomechanical and hemocompatible properties of the resulting functionally active vascular prostheses.

## Materials and Methods

### Manufacturing of functionally active vascular prostheses.

 Vascular prostheses with pro-angiogenesis components were prepared by emulsion electrospinning from a mixture of PHBV and PCL (Sigma-Aldrich, USA). The recombinant human growth factors VEGF and bFGF (Sigma-Aldrich), as well as the recombinant human chemoattractant SDF-1α (Sigma-Aldrich) were also incorporated into the vascular prostheses. The inner layer (1/3 of the wall thickness) of the prosthesis was made from a polymer solution containing 5% PHBV and 10% PCL in trichloromethane mixed (in a ratio of 20:1) with a VEGF solution in physiological saline (10 μg/ml) using a magnetic stirrer to obtaining an emulsion. For the outer layer of the prosthesis (2/3 of the wall thickness), the above polymer solution was mixed (in a ratio of 20:1) with a solution containing equal amounts of bFGF (10 μg/ml) and SDF-1α (10 μg/ml) in physiological saline. The final concentration of each of these biomolecules was 500 ng per 1 ml of polymer solution.

Electrospinning was performed using a Nanon-01A instrument (MECC Inc., Japan) at a voltage of 23 kV, a solution feed rate of 0.5 ml/h, a distance to the collector of 150 mm, and with the help of a blunt 22G needle. A pin of 4 mm in diameter was used as a collector. The wall thickness of the prostheses was 400±46 μm.

### Anti-thrombogenic surface coating of the PHBV/ PCL/GFmix vascular prosthesis.

 Additional surface coating with antiplatelet agents and anticoagulants was performed according to an original method [14]. To this end, a polymer layer was formed on the surface of the earlier made vascular prostheses. First, the samples were soaked in a 5% alcohol solution of polyvinylpyrrolidone (PVP; PanReac, Germany) and air-dried at room temperature for 24 h. Then the vascular prostheses were placed in glass test tubes, gassed with argon, and hermetically sealed with Parafilm. The PVP polymer was grafted onto the surface of the vascular prosthesis under ionizing radiation at a dose of 20 kGy for 2.5 h. In this irradiation regimen, the vascular prostheses got sterilized; therefore, further processing was carried out under sterile conditions. The non-grafted polymer was washed out with sterile water for 1 h.

Considering the ability of PVP to form chemical complexes, subsequent modification was carried out using unfractionated heparin — a direct anticoagulant (solution No.1), and iloprost — an antiplatelet agent — in combination with heparin (solution No.2). The modifying solutions were prepared in glycine buffer (pH 2.61): solution No.1 — 25,000 IU of heparin in 100 ml of buffer; solution No.2 — 12,500 IU of heparin and 20 μg of iloprost in 100 ml of buffer. The vascular prostheses were kept in these solutions for 30 min, dried in air for 24 h, and placed in sterile storage containers.

### Study of physical and mechanical properties.

 The mechanical properties of PHBV/PCL/GFmix vascular prostheses without and with anti-thrombogenic drug coating were studied under conditions of uniaxial tension in accordance with the GOST 270-75. Punching of the samples was carried out in the longitudinal direction of the vascular segment using a specially shaped cutter. The indices of the human internal thoracic artery (a.  mammaria interna), which is considered the gold standard for coronary artery bypass grafting, were used as controls. Segments of the a. mammaria interna were collected during bypass surgery from patients who signed an informed consent agreement for vascular material collection.

The tests were carried out using a universal testing machine of the Z series (Zwick/Roell, Germany) with a sensor of a nominal force of 50 N, with an error of ±1%, and a crosshead movement of 50 mm/min. The ultimate tensile strength of the material was calculated as the maximum extension stress (MPa) required for breaking the sample. Since the thickness of the a. ammaria segments (260±40 μm) was significantly less than that of polymer grafts, we used an additional criterion of strength; that was the maximum force the sample could withstand before breaking down (*Fmax*, N). The elastic deformation characteristics of the sample were assessed by its relative elongation (%) before braking and by the Young’s modulus (MPa); both parameters were determined within the range of physiological pressure (80–120 mm Hg).

### Assessment of hemocompatibility of vascular prostheses modified with heparin and hepariniloprost combination

#### Hemolysis.

Red blood cell rupture resulted from the contact between the blood and the polymer surface was measured according to the ISO 10993.4 standard. To assess the degree of hemolysis, fresh donor blood was added with 3.8% sodium citrate in a ratio of 1:9 (citrate and blood). Five samples sized at 25 cm^2^ each were placed in weighing cups containing 10 ml of physiological solution and incubated for 120 min at 37°C. Saline and distilled water were used as positive and negative controls, respectively. Two hours after the incubation, 200 μl of citrated blood was added to each cup and further incubated for 1 h at 37°C. Then, the polymer samples were transferred from the cups into test tubes and centrifuged for 10 min at 2800 rpm to sediment the remaining cells. Optical density of the obtained supernatants was measured using a GENESYS 6 spectrophotometer (Thermo Scientific, USA) at a wavelength of 545 nm.

The percentage of hemolysis (*H*) was determined by the formula [15, 16]:


$$H=\frac{D_{t}-D_{ne}}{D_{pe}-D_{ne}}\cdot 100\%,$$

where *D_t_* is the optical density of the sample incubated with the test material; *D_ne_* is the optical density of the positive control; *D_pe_* — optical density of the sample after 100% hemolysis.

Zero hemolysis was defined as the mean optical density of saline-diluted blood (*D_ne_*=0). For 100% hemolysis, the optical density of blood added with distilled water was taken (*D_pe_*=0.279).

#### Platelet aggregation.

The study was carried out according to the ISO 10993.4 standard. To obtain platelet-rich plasma (PRP) fresh citrated blood was centrifuged for 10 min at 1000 rpm. Platelet-poor plasma (PPP) was obtained by repeated centrifugation of PRP for 20 min at 4000 rpm. PPP was used to calibrate the instrument. Intact PRP was used as a positive control.

The measurements were carried out in a spontaneous mode without aggregation inductors. To restore the level of Ca^+2^ in citrated blood, CaCl_2_ (0.025 mol/L) was used. The reagent mixture contained 250 μl of PRP and 25 μl of CaCl_2_. The contact time of the test samples with PRP was 3 min, after which the measurements were made.

The maximum aggregation of platelets was measured with a semi-automatic 4-channel platelet aggregation analyzer APACT 4004 (LABiTec, Germany).

#### Platelet adhesion.

The method for assessing the adhesion of blood platelets to the surface of the test materials is based on a visual analysis of platelet deformation, as well as on platelet count [15].

Samples of the studied matrices (3 samples in each group) with a size of 0.5 cm^2^ each were incubated in 300 μl of PRP obtained by the above method. Incubation was carried out for 2 h at 37°C, after which the samples were washed with phosphate buffered saline (PBS, pH  7.4) to remove non-adsorbed plasma components. Then the samples were fixed in a 2% glutaraldehyde for 2 h, then washed again in PBS and dehydrated in a series of alcohols of ascending concentrations from (30, 50, 70, 80, and 100%) for 15 min in each followed by drying at room temperature. The treated samples were mounted on special pads using a carbon tape; a conductive gold-palladium coating was formed on the surface of the samples using an EM ACE200 vacuum instrument (Leica Microsystems GmbH, Austria).

Evaluation of platelet adhesion was carried out using an S-3400N scanning electron microscope (Hitachi, Japan) at an accelerating voltage of 5 kV under high vacuum conditions. For the study, 9 most characteristic fields were randomly selected. The adhesive potential of the tested materials was assessed by the platelet deformation index, i.e., the degree of their transformation from type I (spherical) to type V (absolutely flat). The index was calculated using the formula [15–17]:

Deformation index = (number of platelets type I **·** 1 + +  number of platelets type II  **·** 2 + number of platelets type III **·** 3 + number of platelets type IV **·** 4 + +  number of platelets type V  **·** 5) / total number of platelets.

#### Surface structure.

Evaluation of the surface structure of the tested PHBV/PCL/GFmix prostheses before and after anti-thrombogenic drug coating was carried out using an S-3400N scanning electron microscope at an accelerating voltage of 5 kV under high vacuum conditions. The samples were mounted on special pads using a carbon adhesive tape; then a conductive gold-palladium coating was formed on their surface with the help of an EM ACE200 machine (Leica Microsystems GmbH, Austria).

### Statistical analysis.

 The data were processed using the Prism 8.0 software (GraphPad, USA). The data distribution in the samples was assessed using the Kolmogorov–Smirnov test. The results are presented as the medians (Me) and 25–75 percentiles. The statistical significance of the differences between two independent groups was assessed with the Mann–Whitney U test. Differences were considered significant at a significance level of p<0.05.

## Results

No significant differences in the tension values were found between a. mammaria and the polymer vascular grafts regardless of their modification (p>0.05) (Table 1). It should be noted that with a graft thickness exceeding that of a. mammaria by 1.5 times, the force required to break these vascular prostheses was twice higher than that for a. mammaria (p<0.05). This result indicates that the PHBV/PCL vascular prostheses are superior in terms of strength. Considering that a polymer vessel, unlike a natural one, is not capable of renewal, with an adequate selection of thickness, it will have a certain margin of safety for functioning in the human body.

**Table 1:** Mechanical properties of PHBV/PCL/GFmix vascular prostheses before and after anti-thrombogenic coating; mechanical properties of a. mammaria are shown for comparison (Me [25; 75])

Indicators	PHBV/PCL/ GFmix	PHBV/PCL/ GFmix + PVP + + heparin	PHBV/PCL/GFmix + + PVP + heparin + + iloprost	a. mammaria
Tension (MPa)	3.05	2.66	3.94	2.48
	[2.90; 3.20]	[1.69; 3.81]	[3.78; 3.99]	[1.36; 3.25]
*Fmax* (N)	2.30	2.52	3.08	0.96
	[2.20; 2.50]^+^	[2.05; 2.93]	[2.93; 3.30]*^+^	[0.67; 2.50]
Relative elongation (%)	121.70	59.46	109.17	29.72
	[117.10; 129.60]^+^	[45.60; 87.48]*^+^	[92.29; 116.06]^+^	[23.51; 39.62]
Young’s modulus (MPa)	8.60	28.70	49.95	2.42
	[8.0; 9.64]^+^	[26.0; 36.50]*^+^	[44.90; 54.70]*^+^	[1.87; 3.19]

Note. Statistical significance of the differences (p<0.05): * vs PHBV/PCL/GFmix; ^+^ vs a. mammaria.

After modification of the PHBV/PCL/GFmix vascular prostheses with heparin, or with heparin + iloprost, an increase in the Young’s modulus was found, indicating a greater stiffness of these grafts compared to the unmodified controls (p<0.05).

Vascular prostheses made of PHBV/PCL/GFmix  +   +  PVP  +  heparin  +  iloprost required a significantly higher force to break the sample, and had a stiffness of 1.7   times higher compared to the PHBV/PCL/GFmix + PVP + heparin grafts and 5.8 times higher than the PHBV/PCL/GFmix prostheses, and 20.6 times higher than that of a. mammaria (p<0.05). The relative elongation of the polymer grafts exceeded that of a. mammaria more than 2-fold (p<0.05).

The above results showed a positive effect of the heparin and heparin  +  iloprost modification on the mechanical properties of PHBV/PCL/GFmix vascular prostheses.

After the blood contacted with PHBV/PCL/GFmix prostheses (with or without a drug coating), the degree of hemolysis did not exceed 2% (Table 2), which indicated a high hemocompatibility of all studied samples [18]. There were no significant differences between grafts modified with heparin vs heparin + iloprost. When comparing the indices of vascular prostheses with and without a drug-coating, a significant increase in hemolysis was found in the coated prostheses (p<0.05), but its level did not rise beyond the acceptable 2%.

**Table 2:** Hemolysis and platelet aggregation upon blood contact with polymer grafts (%) (Me [25; 75])

Graft type	Degree of hemolysis	Maximum platelet aggregation
Intact platelet-rich plasma	—	14.61 [13.63; 17.72]
PHBV/PCL/GFmix	0 [0; 0]	17.25 [16.30; 17.96]
PHBV/PCL/GFmix + PVP + heparin	0.36 [0.36; 0.72]*	26.78 [25.47; 31.53]*^+^
PHBV/PCL/GFmix + PVP + heparin + iloprost	0.36 [0.36; 0.36]*	8.22 [8.13; 8.78]*^+^

Note. Statistical significance of the differences (p<0.05): * vs PHBV/PCL/GFmix; ^+^ vs a. mammaria.

The maximum platelet aggregation in intact PRP (positive control) was 14.61 [13.63; 17.72]%. An almost twofold increase in this value was found with the PHBV/PCL/GFmix + PVP + heparin vascular prostheses (p<0.05), while the non-coated PHBV/PCL/GFmix samples had no significant differences vs intact PRP (see Table 2). The PVP + heparin + iloprost coating of PHBV/ PCL/GFmix grafts made it possible to decrease the platelet aggregation by 2.1 and 3.3 times in comparison with the unmodified grafts and the PHBV/ PCL/GFmix + PVP + heparin prostheses, respectively, as well as by 1.8 times — in comparison with intact PRP (p<0.05).

Following the contact with blood, platelets, and plasma proteins were noted on the surface of PHBV/PCL/GFmix grafts with and without anti-thrombogenic coating (Table 3).

**Table 3:** Platelet deformation index and distribution of platelets by type upon the exposure to the surface of polymer grafts

Graft type	Platelet type (%)					Deformation index Me [25; 75]
	I	II	III	IV	V	
PHBV/PCL/GFmix	0	15.4	73.1	9.6	1.9	2.70 [1.0; 3.0]
PHBV/PCL/GFmix + PVP + heparin	4.3	7.8	75.0	12.9	0	2.63 [1.67; 3.14]
PHBV/PCL/GFmix + PVP + heparin + iloprost	0	0	0	0	0	0 [0; 0]*

* p<0.05 as compared with the PHBV/PCL/GFmix graft values.

The surface structure of the PHBV/PCL/GFmix polymer grafts was a porous matrix with fibers of different thicknesses resulted from the growth factors present in the aqueous phase of the initial solution for electrospinning. Modification with heparin and heparin + iloprost did not affect the structure of the initial polymer matrix (see the Figure).

**Figure:**
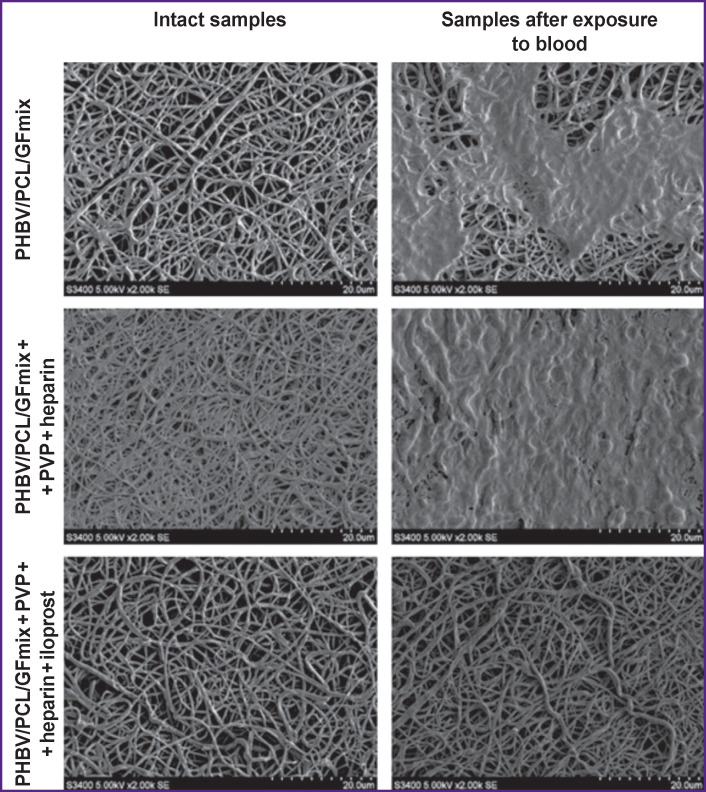
Platelet adhesion on the inner surface of PHBV/PCL/GFmix grafts modified with heparin or heparin + iloprost; ×2000

After contacting the blood, the surface of PHBV/PCL/GFmix + PVP + heparin  + iloprost prostheses was not found to attract platelets or blood proteins. In contrast, type III platelets predominated on the surface of PHBV/ PCL/GFmix and PHBV/PCL/GFmix + PVP + heparin prostheses (see Table 3, the Figure). Despite that the maximum platelet aggregation on the surface of grafts with unfractionated heparin was twice higher than that on the surface of non-heparinized grafts, these surfaces did not significantly differ by the deformation index.

## Discussion

Blood vessel grafts must have high thrombotic resistance. Most often, the risk of thrombus formation is higher in implants intended for the reconstruction of small-diameter vessels, which is associated with the hemodynamics and rheology in this part of the bloodstream. An increase in thrombotic resistance of vascular implants is achievable by modifying their inner surface with antiplatelet agents and anticoagulants [19].

Today, small-diameter tissue-engineered vascular grafts represent a promising alternative to synthetic blood vessels. However, there is a problem to create optimal combinations of the given materials; it is necessary to evaluate the structure of the prosthesis surface (which often imitates the natural extracellular matrix) for its ability to form a vascular tissue. These factors can reduce the thrombotic resistance of newly created structures and call for enhancing the anti-thrombogenic qualities of such implants [19].

In this study, two variants of surface modification of PHBV/PCL/GFmix vascular prostheses were tested: using low-molecular-weight heparin and a combination of low-molecular-weight heparin with iloprost. Modification of the prosthesis with antithrombotic agents is carried out by forming a PVP hydrogel coating on its inner surface to bind anti-platelet agents and anticoagulants. In addition, the formation of a PVP hydrogel layer increases the hydrophilicity of the prosthesis surface, which helps reduce the adhesion of proteins and blood cells, in particular platelets, as well as prevent conformational changes of proteins. The speedy desorption of proteins — an additional anti-thrombogenic factor — is due to the mobility of macromolecular chains in hydrogels.

Grafting PVP to the prosthesis surface was performed using the method of radiation-induced grafting polymerization; in this procedure, the product is simultaneously sterilized by ionizing radiation. In an irradiated sample, the bioactivity of differentiation factors incorporated into the prosthesis is preserved for a long time. After the procedure, the subsequent attachment of drugs through complexation with PVP leads to the formation of a drug coating in which the drug component partially penetrates into the PVP polymer and partially remains in the lumen of the vascular prosthesis. Due to this, when the prosthesis comes into contact with the blood, the anti-thrombogenic effect of the drugs occurs instantly. This method of drug incorporation, in contrast to covalent binding, allows them to preserve their biological activity without creating steric obstacles and without blocking the interaction of the drugs with blood coagulation factors.

With the combined use of unfractionated heparin and iloprost, a significant increase in the anti-thrombogenic power of the PHBV/PCL/GFmix prostheses was achieved.

## Conclusion

The technology of anti-thrombogenic surface coating of biodegradable porous vascular PHBV/PCL/GFmix prostheses with the combined use of unfractionated heparin and iloprost significantly improves the hemocompatibility of the prostheses; this manifests in the absence of platelet and blood protein adhesion to the surface of the prostheses. Ionizing irradiation triggering the polyvinylpyrrolidone cross-linking on the PHBV/PCL/GFmix surface increases the stiffness and strength of the final product.
